# Challenging wide QRS tachycardia diagnosis: One trigger two mechanisms

**DOI:** 10.1016/j.ipej.2024.04.008

**Published:** 2024-05-01

**Authors:** Oğuzhan Ekrem Turan, Barış Akdemir, Reşit Yiğit Yilancioğlu, Emin Evren Özcan

**Affiliations:** a Dokuz Eylul University, Faculty of Medicine, Heart Rhythm Management Center, 35220, Izmir, Turkey; b Yeni Yuzyil University, Faculty of Medicine, Cardiology Department, Turkey

**Keywords:** Accessory pathway, Atriofasicular, Mahaim, Ventricular arrhythmia, Antidromic

## Abstract

The coexistence of different types of wide QRS complex tachycardias induced by the same trigger has rarely been observed. The electrical instability and incessant nature of tachycardias can cause tachycardiomyopathy and will not allow accurate diagnosis during an electrophysiological study (EPS). In case of an electrical storm, elimination of the trigger may be the first approach to provide patient stability. We report a successfully managed case of repetitive initiation of pleomorphic ventricular tachycardia and Mahaim-type antidromic atrioventricular reentrant tachycardia, induced by a premature ventricular complex in the right ventricular outflow tract.

## Abbreviations

WCT: Wide QRS complex tachycardia
ECG: Electrocardiogram
PVC: Premature ventricular complex
RVOT: Right ventricular outflow tract
HRA: High right atrium
CS: Coronary sinus
RVA: Right ventricular apex
MAP: Mahaim-type atriofascicular accessory pathway
AVNRT: Atrioventricular nodal reentrant tachycardia
AT: Atrial tachycardia
HV: His-ventricular
LBBB: Left bundle branch block
EPS: Electrophysiological study

## 1 Introduction

Recognition of differential diagnosis of wide QRS complex tachycardia (WCT) is crucial during acute management strategies. The most critical step is to determine whether tachyarrhythmia has ventricular or supraventricular origin. ECG interpretation is important, but it may not always provide sufficient information. The mechanism of wide complex tachycardia may be ventricular tachycardia, supraventricular tachycardia with preexisting or functional aberrancy, and preexcitation syndrome or the combination of those [1]. We report a case with pleomorphic ventricular tachycardia (VT) and Mahaim-type antidromic atrioventricular reentrant tachycardia (AVRT) induced by right ventricular outflow tract (RVOT) premature ventricular complex (PVC).

## 2 Case presentation

**Fig. 1:**
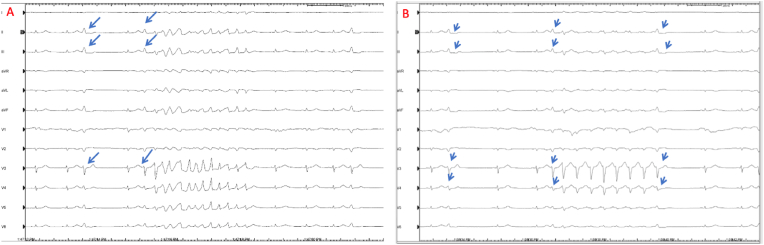
**A**) Pleomorphic VT triggered by RVOT PVC (Blue arrow) **B)** Mahaim Type AVRT triggered and stopped by same PVC (Blue arrow).

A 46-year-old female patient was admitted for a WCT. The patient was on an antiarrhythmic drug, flecanide, at an external centre because of frequent premature ventricular beats. The patient was hemodynamically stable, although different types of WCTs were observed (Fig. 1A and B) Bigeminy PVC was observed before the WCT episodes. Physical examination did not reveal any significant findings except for irregular and fast heartbeats. Echocardiography revealed moderately reduced left ventricular ejection fraction and global hypokinesia, consistent with tachycardiomyopathy. Coronary angiography revealed no obstruction. The patient was scheduled for catheter ablation.

**Fig. 2:**
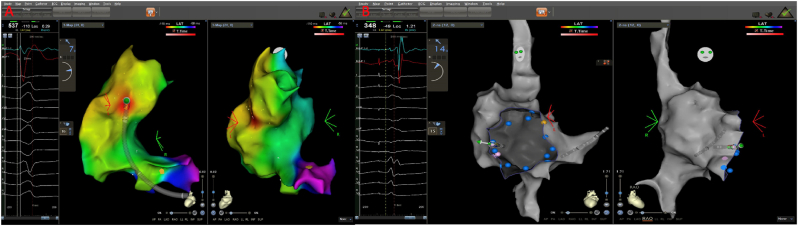
**A)** 3D reconstruction of the RVOT (left) and right atrium-tricuspid annulus (right). PVC was ablated from the posterior septal portion of the RVOT. **B)** The Mahaim-type atriofascicular pathway was located on the right atrial free wall of the tricuspid annulus (9 o'clock) and ablated from the atrial insertion to the lateral tricuspid annulus.

**Fig. 3:**
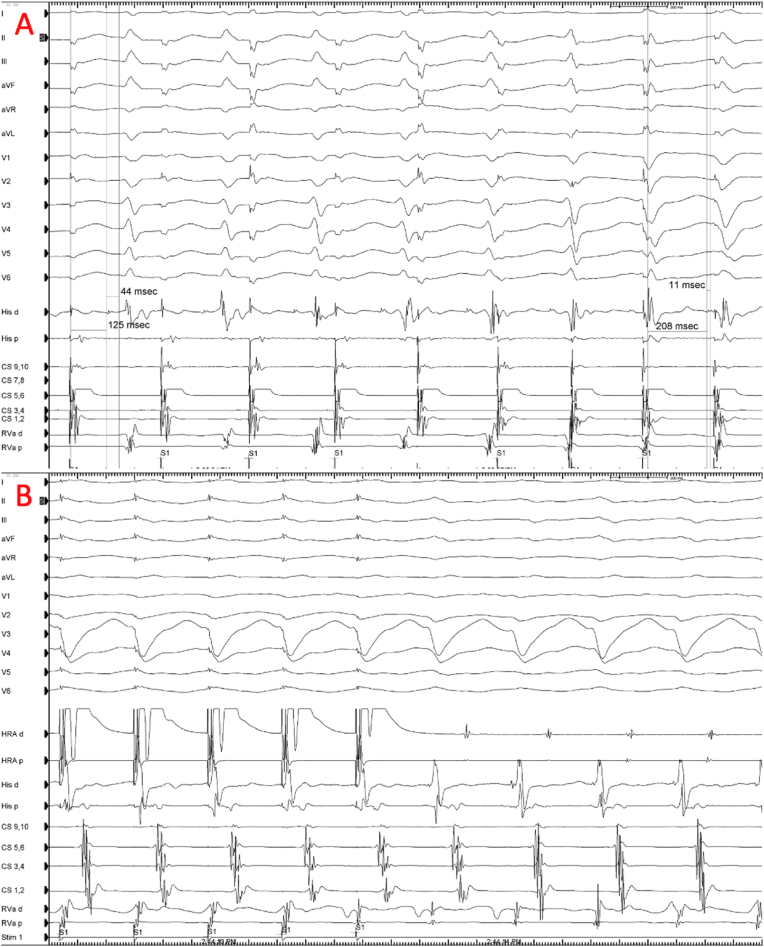
**A) Incremental pacing from CS** (coronary sinus) 5–6 electrode revealed the prolongation of AH interval and shorten of HV. QRS morphology progressively widened during atrial pacing. RVa (right ventricular apex) catheter electrocardiogram precedes His catheter **B)Findings of Atrial Entrainment in tachycardia**: HV-negative tachycardia was observed. This finding excludes SVT with aberrancy. Atrial entrainment with conceald fusion during wide QRS tachycardia and obtaining an A-V-A response excluded myocardial VT. VA-linking was present in atrial entrainment which is exclude AT in responsible mechanism of tachycardia Occurrence of right ventricular apex activation before the His and normal HV interval at baseline most likely excludes bundle branch reentrant VT.

In the electrophysiological study, catheters were placed in the high right atrium (HRA), coronary sinus (CS), and right ventricular apex (RVA). Programmed atrial and ventricular stimulation could not be performed because of the repetitive initiation of pleomorphic VT (Fig. 1A). The ablation strategy was first set up to abolish the constant visible RVOT morphology of PVC, which triggered the WCTs. Endocardial mapping was performed using a smart-touch contact force (CF)-sensing catheter (Biosense Webster, Inc., CA, USA) with the CARTO 3 v7.1 mapping system (Carto, Biosense Webster). The earliest PVC activation site was the posteroseptal portion of the RVOT (Fig. 2A). After eliminating the PCVs, pleomorphic VT ceased. Programmed ventricular extrastimuli did not induce VT and revealed retrograde decremental conduction properties compatible with AV nodal response. Atrial incremental pacing showed progressive lengthening of the A–H interval, contemporary shortening of the H–V interval, and progressive preexcitation of QRS complexes with LBBB morphology (Fig. 3A). The tachycardia with LBBB morphology was induced by ventricular extrastimuli. A negative HV during tachycardia excludes LBBB aberrancy with AVNRT and/or AT. Ventricular tachycardia was excluded by atrial entrainment with concealed fusion and A-V-A response (Fig. 3B). Mahaim-type atriofascicular accessory pathway (MAP) related AVRT was diagnosed. MAP was localised at the right atrial free wall of the tricuspid annulus (9 o'clock) and was ablated from the atrial insertion (Fig. 2B). After ablation of the Mahaim fiber, no sustained tachycardia was induced and no pleomorphic VT was observed under isoprenaline infusion. The patient had no arrhythmias during one year of follow-up. In addition, the patient's ejection fraction was normal and there were no abnormalities on MRI which aimed to investigate other underlying pathologies.

## 3 Discussion

Differential diagnosis of wide QRS tachycardia is challenging, and even more difficult, when the patient has more than one wide complex tachycardia. Moreover, a single trigger can reveal different mechanisms that initiate multiple arrhythmias and electrical instabilities. Eliminating triggers in alternating electrical storm situations provides electrical stability and enables diagnostic maneuvers for accurate diagnosis.

The irregularity observed during WCT can be explained by automaticity, triggered activity-induced ventricular tachycardia, or pre-excited atrial fibrillation [2]. Another rare mechanism is the AV node-like accessory pathway automaticity. MAPs may have their own automaticity, which is typically observed during ablation [3]. Theoretically spontaneous irregular pleomorphism can occur as an intra-Mahaim tachycardia; however, this has not been reported in the literature.

Repetitive and non-sustained initiation of WCT by PVCs can complicate the management of cases. Observation during the WCT is the first crucial step in diagnosis and management. Maneuvers during EPS are used for accurate diagnosis. The differential diagnosis of WCT may not be easy during EPS because of the continuous reinitiation of tachycardia. Identifying the first target and abolishing the trigger may allow maneuvers to be performed, thus making it easy to reveal the main mechanism. Sometimes frequent PVCs can be combined with NSVT or supraventricular tachycardia (SVT); these manifestations are commonly benign, whereas malignant PVC-induced ventricular arrhythmias such as ventricular fibrillation or polymorphic/pleomorphic ventricular tachycardia are rarely reported in the literature [4]. In a study on this subject by Takashi Noda et al. no cause, such as QT interval, coupling interval, or other ECG changes, could be found to cause malignant behavior in most cases, as in our case. Hypothetically, this could be due to a functional block caused by MAP-associated conduction and the resulting micro-reentry. Therefore, we aimed to abolish outflow tract origin PVC, which might trigger different types of WCTs. Pleomorphic VT was no longer induced after ablation of the RVOT PVC.

MAP reentrant tachycardia should be considered when WCT has left bundle branch block (LBBB) morphology. Tachycardia-induced cardiomyopathy caused by MAP-related reentrant tachycardia is rarely observed but has a good prognosis after ablation [5]. Also, frequent PVCs and ventricular arrhythmias contribute to the development of cardiomyopathy [6]. Trigger elimination can suppress electrical storms and contribute to the resolution of cardiomyopathy [7]. In our case, we also performed cardiac MRI and did not find any underlying diseases such as ARVD.

## 4 Conclusion

PVCs can cause cardiomyopathies and electrical instability which result in ventricular tachycardia. Trigger elimination is important to achieve electrical stability and enable accurate diagnosis.

## Data availability statement

The data that support the findings of this study are available from the corresponding author, upon reasonable request.

## Funding statement

None.

## Patient consent statement

The authors confirm that written consent for the submission and publication of this case, including images, was obtained from the patient in line with the COPE guidance.

## Ethical statement and informed consent

Written informed consent was obtained from participant in this case.

## Statement of consent

All authors declare that no conflict of interest.

## Funding

No funding

## Declaration of competing interest

The authors declare that they have no known competing financial interests or personal relationships that could have appeared to influence the work reported in this paper.
